# Genome-Wide Identification, Characterization and Expression Profiling of *myosin* Family Genes in *Sebastes schlegelii*

**DOI:** 10.3390/genes12060808

**Published:** 2021-05-25

**Authors:** Chaofan Jin, Mengya Wang, Weihao Song, Xiangfu Kong, Fengyan Zhang, Quanqi Zhang, Yan He

**Affiliations:** 1 MOE Key Laboratory of Molecular Genetics and Breeding, College of Marine Life Sciences, Ocean University of China, Qingdao 266003, China; 18663360681@163.com (C.J.); Mengya0828@163.com (M.W.); songweihao8@163.com (W.S.); 15621456335@163.com (X.K.); YyZhang0109@163.com (F.Z.); qzhang@ouc.edu.cn (Q.Z.); 2 Laboratory of Tropical Marine Germplasm Resources and Breeding Engineering, Sanya Oceanographic Institution, Ocean University of China, Sanya 572000, China; 3 Laboratory for Marine Fisheries Science and Food Production Processes, Qingdao National Laboratory for Marine Science and Technology, Qingdao 266003, China

**Keywords:** *myosin* gene family, *myo2*, skeletal muscle growth, myoblast differentiation, *Sebastes schlegelii*

## Abstract

Myosins are important eukaryotic motor proteins that bind actin and utilize the energy of ATP hydrolysis to perform a broad range of functions such as muscle contraction, cell migration, cytokinesis, and intracellular trafficking. However, the characterization and function of *myosin* is poorly studied in teleost fish. In this study, we identified 60 *myosin* family genes in a marine teleost, black rockfish (*Sebastes schlegelii*), and further characterized their expression patterns. *myosin* showed divergent expression patterns in adult tissues, indicating they are involved in different types and compositions of muscle fibers. Among 12 subfamilies, *S. schlegelii myo2* subfamily was significantly expanded, which was driven by tandem duplication events. The up-regulation of five representative genes of *myo2* in the skeletal muscle during fast-growth stages of juvenile and adult *S. schlegelii* revealed their active role in skeletal muscle fiber synthesis. Moreover, the expression regulation of *myosin* during the process of myoblast differentiation *in vitro* suggested that they contribute to skeletal muscle growth by involvement of both myoblast proliferation and differentiation. Taken together, our work characterized *myosin* genes systemically and demonstrated their diverse functions in a marine teleost species. This lays foundation for the further studies of muscle growth regulation and molecular mechanisms of indeterminate skeletal muscle growth of large teleost fishes.

## 1. Introduction

Myosins are a large family of cytoskeletal motor proteins that bind filamentous actin and utilize the energy of ATP hydrolysis to play an important part in divergent biological process, such as muscle contraction, cell motility and contractility, cytokinesis, and intracellular trafficking [1,2,3]. *Myosin* was firstly described in rabbits [4]. Subsequently, cardiac muscle and smooth muscle *myosin* was reported in studies conducted by Bailey and Cohen et al. [5,6]. Myosins are typically composed of three domains, a conserved head located in the N-terminal that binds to actin filaments, a short neck as a binding site for myosin light chain, and a tail located in the C-terminal generally binding to the motor “cargo” to determine the functions of the motor [7,8]. Eukaryotes contain up to 35 *myosin* subfamilies based on an analysis of 2269 Myosin motor domains from 328 organisms [9]. Generally, the *myo2* subfamily, as known as *myosin 2* (myosin heavy chain, *MYH* or *MHC*), is considered to be the conventional *myosin*, which is the main component of skeletal muscle whereas the other *myosin* subfamilies are considered to be unconventional *myosin* [10].

*Myosin* family genes have been widely identified to be functional in multiple biological process [2]. For conventional *myosin*, *MYH2* and *MYH7* have been shown to play an important role in the formation of muscle fibers and are stably expressed in different types of muscle fibers [11]. *MYH11* is proven to be a useful marker to define myoid cells in mouse testis [12]. For unconventional *myosin*, *myosin 3* plays a key role in regulating stereocilia lengths required for normal hearing [8]. *myosin 5* is essential in intracellular transport of organelles, mRNA and other cargo [13]. Among the many functions of *myosin* genes, their role in the organization of muscle fibers is best characterized, as described in mammal, shrimp, and silkworm [14,15,16]. A recent study reported that knocking down of myosin heavy chain caused the inhibition of sarcomeric organization of thin filaments in larval musculature in oysters, suggesting the vital role of *MYH* in muscle development of aquatic economic species [17].

In the aquaculture industry, skeletal muscle growth is closely related to the growth trait of cultured fish, which will directly impact the economic benefit. Over the past decade, multiple strategies have been applied to improve the fish production, including genetic engineering breeding and interspecific hybridization [18]. Illustrating the molecular mechanism of skeletal muscle growth will contribute to making strategies to promote the growth of cultured fish. One early study was carried out to demonstrate the *MYH* classification, evolution, and expression in fish [19]. More recent studies focused on some specific members of *myosin* and characterized their expression and function in fish [20,21,22,23]. Despite the great process of characterization of *myosin* genes in teleost fish, the genome-wide phylogenetic identification and characterization of complete *myosin* family genes in teleost is still poorly conducted.

Black rockfish (*Sebastes schlegelii*), distributing in China, Japan, and Korea, is an economic important marine teleost species. The postnatal growth of *S. schlegelii* exhibit an indeterminate growth pattern, involving both the recruitment and hypertrophy of muscle fibers [24]. The availability of chromosome-level genome sequence [25] provides us the opportunity to analyze *myosin* genes in genome-wide scale. In this study, we identified and characterized 60 *myosin* genes in *S. schlegelii* and further characterized their expression patterns. Our work provides valuable information for further detailed functional analysis of *myosin* in the muscle growth of large teleost fish.

## 2. Materials and Methods

### 2.1. Ethics Statement

This study was approved by the College of Marine Life Sciences, Ocean University of China Institutional Animal Care and Use Committee on 10 October 2018 (Project Identification Code: 20181010).

### 2.2. Identification of Myosin Family Genes in S. schlegelii

To identify *myosin* family genes in *S. schlegelii*, the genome and transcriptome database, which are available at CNSA (CNGB Nucleotide Sequence Archive) under the accession ID CNP0000222 were searched against the Myosin amino acid sequences from some representative species, including *H. sapiens*, *M. musculus*, *O. latipes*, *O. niloticus*, *L. oculatus*, and *G. aculeatus*. TBLASTN algorithm search was performed and sequences with the e-value below e^-5^ were collected. Then, the putative *myosin* sequences were used as query to blast 89 transcriptome assembly of *S. schlegelii*. Only the sequences got hits in at least one transcriptome was regarded as the candidate *myosin* genes. Candidate *myosin* family members were submitted to SMART [26] and NCBI-CDD (https://www.ncbi.nlm.nih.gov/Structure/cdd/wrpsb.cgi) database to predict motor domain. The intracellular location of the proteins was predicted by WoLF PSORT (https://www.genscript.com/psort/wolf_psort.html, accessed on 7 January 2021). The chromosome position, coding strand of *myosin* genes was identified based on genome annotation file. Tandem duplication analysis was conducted according to two criteria: (a) the similarity of aligned sequences was > 70%; (b) two genes were located in the same chromosomal region within 100 kb [27,28].

### 2.3. Molecular Characters of S. schlegelii Myosin Genes 

The physiochemical properties of identified *myosin* genes, including the molecular weight (Mw) and theoretical isoelectric point (pl), were calculated by the ExPasy site (https://web.expasy.org/protparam/, accessed on 7 January 2021) [29,30]. The exon-intron structure of *myosin* genes was determined by comparison of genome and transcriptome sequences following the GT-AG rule [31]. The schematic diagram of gene structure was displayed using GSDS 2.0 program (http://gsds.cbi.pku.edu.cn/, accessed on 30 January 2021). Protein motif analysis was carried out with Motif Elicitation (MEME) program (http://meme-suite.org/tools/meme, accessed on 30 January 2021). 

### 2.4. Phylogenetic and Synteny Analysis

Multiple sequences alignments of Myosin proteins were conducted by Clustal W [32]. The Neighbor-Joining method was utilized to perform phylogenetic analysis by MEGA 7.0 [33] and visualized using iTOL website (http://itol.embl.de, accessed on 20 January 2021). The conserved synteny of *myosins* and their adjacent genes among spotted gar, teleost fish species, and mammals was analyzed based on the genomics website (http://www.genomicus.biologie.ens.fr/genomicus-82.01/cgi-bin/phyloview, accessed on 13 May 2021) and the schematic diagram was drawn by hand.

### 2.5. Expression Profiling of Myosin Genes at Early Ddevelopmental Stages, Different Tissues, and During in Vitro Myoblast Differentiation Process

In our previous studies, we built a large number of transcriptome database of *S. schlegelii*, including different tissues and early developmental stages [25]. In this study, a total of 89 transcriptomes, including 63 tissue libraries and 26 early developmental stages were utilized to characterize the expression profiles of *S. schlegelii myosin* genes (Table S1 and Table S2). We also successfully established a continuous skeletal muscle cell line from juvenile rockfish muscle in one of related study. The cell line consisted of a high ratio of myoblasts and could differentiate into myotubes upon differentiation medium treatment. The transcriptomes of muscle cells at different time points after differentiation medium induction were generated and processed (BioProject ID: PRJNA661185) (This data will be published in other study). TPM (Transcripts Per Million reads) value of *myosin* genes were extracted (Table S3). Heat maps were created by TBtools [34]. 

### 2.6. qRT-PCR Validation of Myosin Expression

Total RNA from all collected samples were isolated using TRIzol reagent (Invitrogen, California, USA) according to the manufacturer’s protocol. After removing genomic DNA with DNase I (Takara, Dalian, China), cDNA synthesis was performed with Reverse Transcriptase M-MLV Kit (Takara, Dalian, China). The quantity and quality of cDNA were tested by spectrophotometry and agarose gel electrophoresis (Coolaber, Beijing, China), respectively. Specific primers for real-time PCR were designed using Integrated DNA Technologies (http://sg.idtdna.com/pages/home, accessed on 2 February 2021). qRT-PCR was performed on LightCycler480 (Roche, San Francisco, USA) using the NovoStart® SYBR qPCR SuperMix Plus (Novoprotein, Shanghai, China) in a condition with 95 °C for 5 min, 45 cycles (95 °C for 15 s) and 60 °C for 45 s. The *EIF5A1* gene in *S. schlegelii* was used as a reference gene to normalize the expression of target genes. The relative expression levels of target genes were calculated based on 2^-^^△△Ct^ comparative Ct method. Statistical analysis was conducted using one-way analysis of variance followed by the least significant difference test using SPSS2.0 (IBM, Armonk, NY, USA), and differences with *P* < 0.05 were treated as significant. Each experiment was performed with triplicates.

### 2.7. Histological Examination 

For histological examination, the samples of muscle from juvenile at different days post parturition (20 dpp, 35 dpp, 50 dpp, 75 dpp, and 90 dpp) and adult *S. schlegelii* at two different ages (1.5-year-old and 2.5-year-old) were collected and fixed in 4% paraformal-dehyde (PFA) overnight at room temperature. Then, the samples were transferred to methanol (30%, 50%, 70%, 80%, 90%, 2 h, respectively), and finally stored in 100% methanol. After dehydrating and embedding in paraffin, tissue blocks were sectioned at 6 μm, and stained with hematoxylin and eosin (Solarbio, Beijing, China).

## 3. Results 

### 3.1. Identification of Myosin Genes in S. schlegelii

The Myosin amino acid sequences from some representative species, including *H. sapiens*, *M. musculus*, *O. latipes*, *O. niloticus*, *L. oculatus*, and *G. aculeatus* were used to search against *S. schlegelii* genome and transcriptome database. In total, 60 *myosin* genes in *S. schlegelii* were identified, including 27 conventional *myosin* genes and 33 unconventional *myosin* genes. *S. schlegelii myosin* genes varied largely in length and physicochemical properties. The length of coding sequences of *myosin* genes ranged from 2862 to 11,313 bp, with corresponding protein length ranged from 954 to 3771 amino acids. Based on primary protein sequences, the molecular weights and isoelectric points of Myosins were predicted. The molecular weight of Myosin proteins ranged from 109.70 to 435.08 kDa with the isoelectric point ranging from 5.42 to 9.43. The number of transcripts produced by each gene was also counted. We found that 29 *myosins* had more than one transcript. *myo7b* and *myo9ab* produced the highest number (seven) of transcripts. The detailed information of *myosin* genes was summarized in Table 1. 

### 3.2. The Evolutionary Relationship of Myosin Genes 

**Figure 1:**
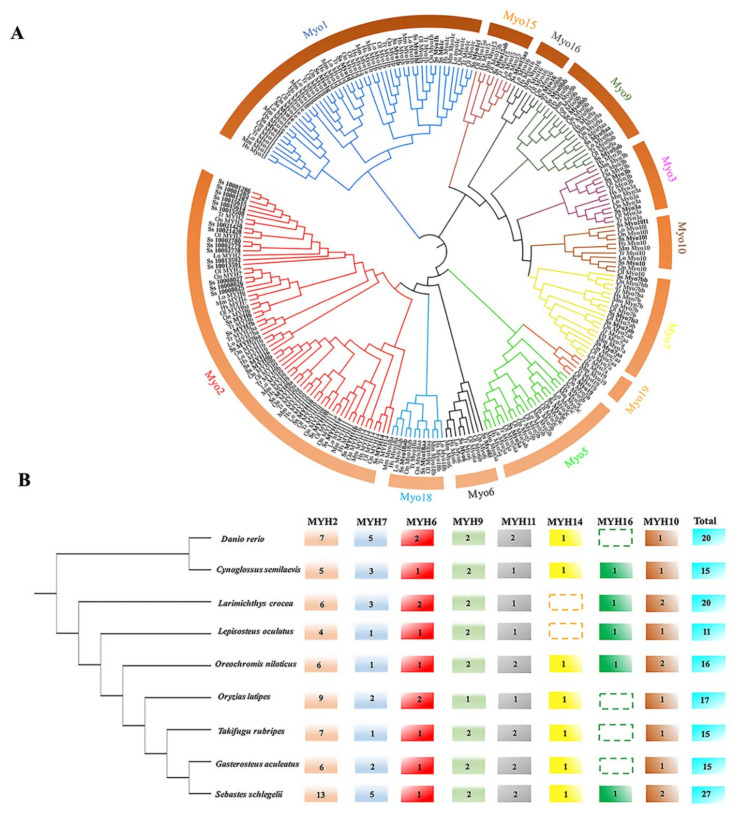
The phylogenetic analysis and classification of *myosin* genes. (**A**) Phylogenetic tree of *myosin* genes was constructed with Neighbor-Joining method by MEGA7 (bootstrap = 1000). Twelve subfamilies were decorated by different colors. (**B**) Characterization of *Myo2* subfamily in teleost fishes. The phylogenetic tree was conducted by MEGA 7.0 using CO1 sequences of these species. The dotted border represented gene loss in the species.

To understand the evolutionary relationship and classification of *myosin*, the phylogenetic analysis was conducted by Neighbor-Joining method. As shown in Figure 1A, 60 *S. schlegelii myosin* were classified into 12 subfamily clusters. *Myo1* and *Myo2* were the first two largest subfamilies in *S. schlegelii*, including 7 and 27 members, respectively. Compared to mammals and other teleost species, *Myo2* subfamily in *S. schlegelii* were significantly expanded (*p* = 0.000003), which could be further divided into eight subfamilies. Among *Myo2* subfamily in *S. schlegelii*, almost half of the genes were clustered with *MYH2* genes from other species, suggesting the expansion of *MYH2* contributed the expansion of *S. schlegelii* conventional *myosin* genes. The member and number of *Myo2* genes in some representative teleost fish were concluded in Figure 1B.

### 3.3. Functional Domains and Gene Structure of Myosin Genes 

**Figure 2:**
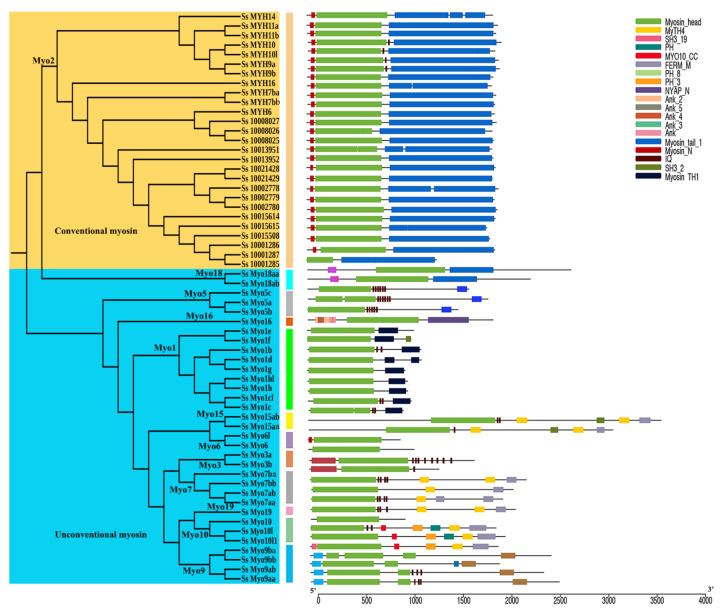
A schematic diagram of *S. schlegelii myosin* gene domains. The maximum likelihood method was used to construct the phylogenetic tree of *myosin* genes (left panel). The rectangle with different colors represented different domains (right panel). The scale bar indicated 500 amino acid residues.

The putative functional domains were identified using SMART and Pfam databases. As shown in Figure 2, all *S. schlegelii myosin* genes had a motor domain (myosin head), which located in the N-terminus. In addition to myosin head, all *Myo2* genes had a Myosin-N domain, except *Ss_10001285*. Furthermore, the domain types and numbers of *myosin* genes were similar within the same subfamily members but divergent among different subfamilies.

**Figure 3:**
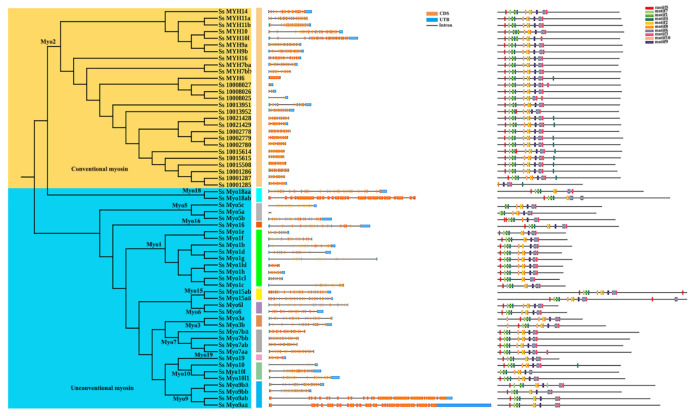
Gene structure and motif analysis of *myosin* genes. The maximum likelihood method was used to conduct the phylogenetic analysis of *myosin* genes (left panel). The exons and UTRs were indicated by orange and blue boxes (the middle panel) respectively. Different putative motifs were indicated by boxes with different colors (right panel).

To gain insights into structures of *myosin* genes, we conducted the exon-intron structure analysis based on genome and transcriptome data following the GT-AG rule (Figure 3, left panel). The genomic structure was divergent among different *myosin* subfamilies. The motif analysis of *S. schlegelii myosin* genes was further performed (Figure 3, right panel). Motif1, motif4, and motif7, locating in myosin head domain, were present in all *myosin* genes. The number and category of motifs were divergent in different subfamilies, but was relatively conserved within the same subfamily. For instance, most *Myo2* subfamily genes shared the same motifs but differed from the motifs conserved in *Myo1* subfamily genes (Figure 3, right panel).

### 3.4. Chromosome Distribution and Synteny Analysis of Myosin Genes

**Figure 4:**
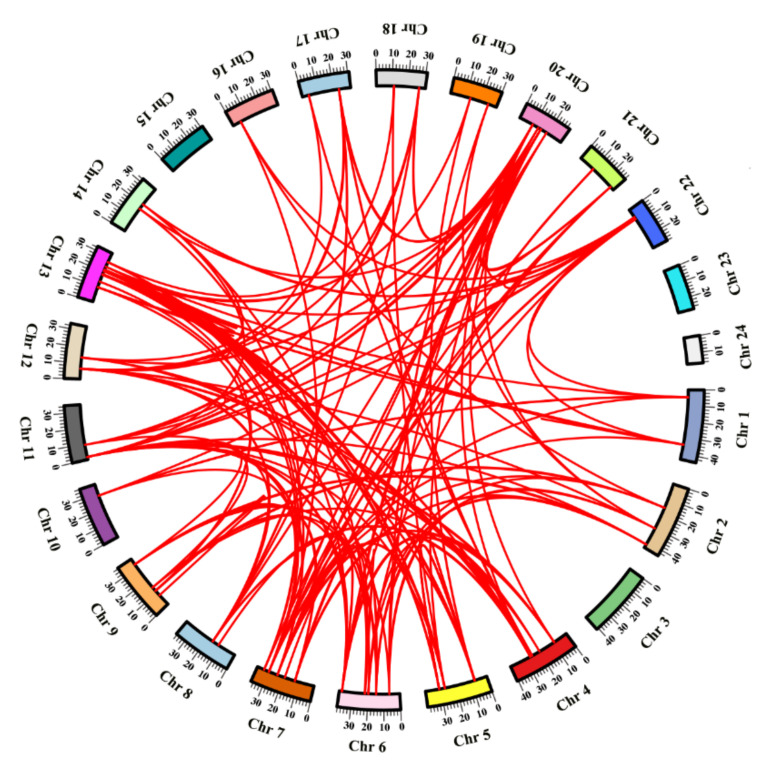
Chromosome location and synteny analysis of *myosin* genes. The boxes with different colors represented different chromosomes of *S. schlegeli*i. The red lines represented the syntenic relationships of *myosin* genes in *S. schlegelii*.

The positions of *myosin* genes on the corresponding chromosomes of *S. schlegelii* were indicated in Figure 4. All *myosin* genes could be mapped onto 21 chromosomes of *S. schlegelii.* Chromosome 13 contained the highest number of *myosin* genes (9), whereas no myosin genes was located on chromosome 15, chromosome 23 and chromosome 24 (Figure 4). To understand the consequences of teleost genome duplication (TGD) on *myosins* evolution, we conducted synteny analysis of *myosins* and their adjacent genes among spotted gar (*Lepisosteus oculatus*), whose lineage diverged before TGD, four teleost fish species (*Gasterosteus aculeatus*, *oryzias latipes*, *Orechromis niloticus* and *S. schlegelii*) and mammals (*Homo sapiens* and *Mus musculus*). We found 14 *myosins* (*myo1hl*, *myo1cl*, *myo6l*, *myo7ab*, *myo7bb*, *myo9ab*, *myo9bb*, *myo10l*, *myo15ab*, *myo18ab*, *MYH7bb*, *MYH9b*, *MYH10l,* and *MYH11*) were generated by TGD whereas the expanded *MYH2* genes specifically emerged in tandem duplication form in *S. schlegelii* (Figure S1). Fifteen *myosin* genes, including three *MYH7* genes and 12 *MYH2* genes were clustered into six tandem duplication event regions on chromosome 1 (one cluster), chromosome 7 (one cluster), chromosome 9 (two clusters), and chromosome 13 (two clusters) (Figure S1 and Table 2).

### 3.5. Expression Patterns of Myosin Genes in Adult Tissues and Early Developmental Stages

**Figure 5:**
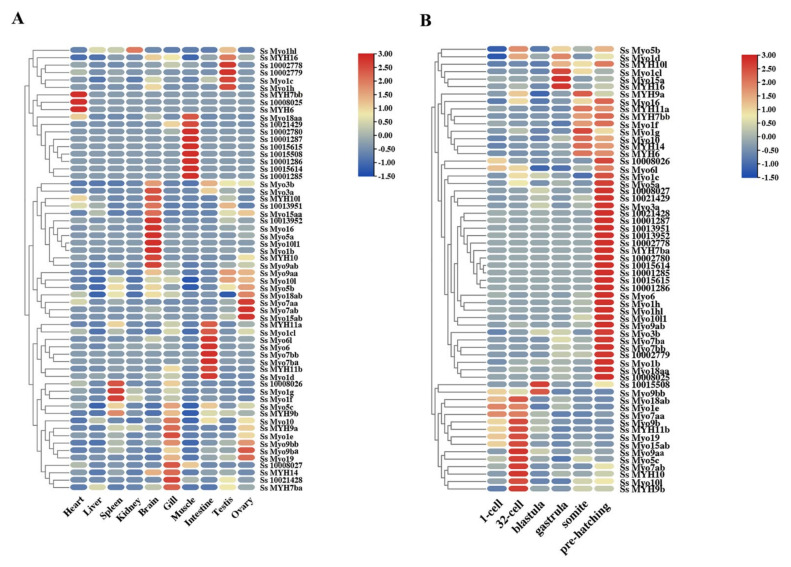
Expression profiles of *myosin* genes in adult tissues (**A**) and early developmental stages (**B**). The color scale represented the TPM (Transcripts-per-million) value. The red and blue color represented the relatively higher and lower TPM (Transcripts-per-million) value, respectively.

The expression levels of *myosin* genes in ten adult tissues (Heart, Liver, Spleen, Kidney, Brain, Gill, Muscle, Intestine, Testis, and Ovary) and six early developmental stages (1-cell, 32-cell, blastula, gastrula, somite, and pre-hatching) were evaluated by TPM (Transcripts per million) values from 89 transcriptome database (Figure 5A). In adult tissues, the expression patterns of *myosin* genes were divergent. For example, seven *myo2* genes (*Ss_10001285*, *Ss_10001286*, *Ss_10001287*, *Ss_10015614*, *Ss_10015615*, *Ss_10015508*, and *Ss_10002780*) were highly expressed in muscles, whereas the rest of 20 *myo2* genes were highly expressed in heart, brain, intestine, and gonads, respectively. In addition to conventional *myosin* genes, some unconventional *myosin* genes also showed tissue-specific expression patterns.

All *S. schlegelii myosin* genes could be detected with the dynamic expression patterns during the early developmental stages (Figure 5B). Most *myosin* genes showed the highest expression level at the pre-hatching stage, including all *MYH2* genes and most other *Myo2* subfamily genes. In addition, some *Myo2* genes showed distinct expression patterns. For instance, the expression of *MYH16* showed the highest level at gastrula, whereas the expression levels of *MYH7b*, *MYH14*, and *MYH6* began to increase at somite stage, and *Ss_10015508* expressed incredibly high at blastula stage.

### 3.6. Myo2 Genes Participate in the Muscle Growth in Juvenile and Adult S. schlegelii 

**Figure 6:**
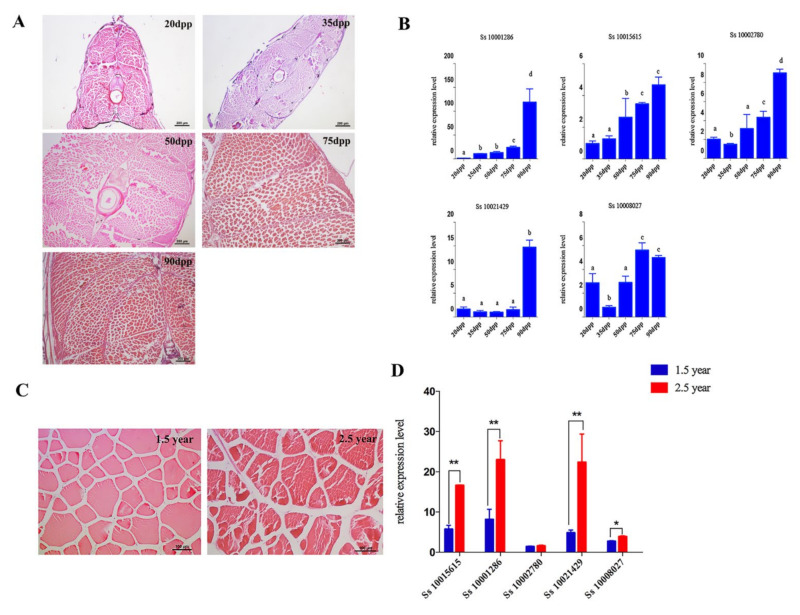
*Myo2* genes participated in *S. schlegelii* muscle growth. (**A, C**) H&E staining showed the process of muscle growth in juvenile and adult *S. schlegelii*. (**B**) Relative expression of five *Myo2* genes during different stages of muscle growth in juvenile fish. Data are shown as mean ± SEM (*n* = 3). Different letters indicated statistical significance (*p* < 0.05). (**D**) Relative expression of five *Myo2* genes in adult (1.5 and 2.5-year-old) *S. schlegelii.* Data are shown as mean ± SEM (*n* = 3). Marks * and ** indicated statistical significance (*p* < 0.05 or *p* < 0.01).

We firstly performed the histological observation of muscle from juvenile *S. schlegelii* at different days post parturition (20 dpp, 35 dpp, 50 dpp, 75 dpp, and 90 dpp). As shown in Figure 6A, the sarcomere gradually widened and muscle fibers gradually became longer and thicker with the growth of *S. schegelli*. 

To further explore the roles of myosin genes in the growth process of *S. schlegelii*, we selected several muscle-highly-expressed and significantly expanded *Myo2* genes (*Ss_10001286*, *Ss_10021429*, *Ss_10015615*, *10002780* of *MYH2* and *Ss_10008027*of *MYH7*), and measured their expression levels in the muscle of juvenile fish. *Ss_10001286*, *Ss_10002780* and *Ss_10021429* were significantly up-regulated from 75 dph to 90 dpp, while the expression levels of *Ss_10015615* and *Ss_10008027* increased since 35 dpp (Figure 6B).

In adult *S. schlegelii*, the diameter and cross-sectional area of muscle fibers increased rapidly from 1.5 to 2.5 years old (Figure 6C). Except for *Ss_10002780*, the other four selected genes showed an extremely significant increase of expression in 2.5-year-old fish (Figure 6D).

### 3.7. Myosin Genes Involved in Myoblast Differentiation 

**Figure 7:**
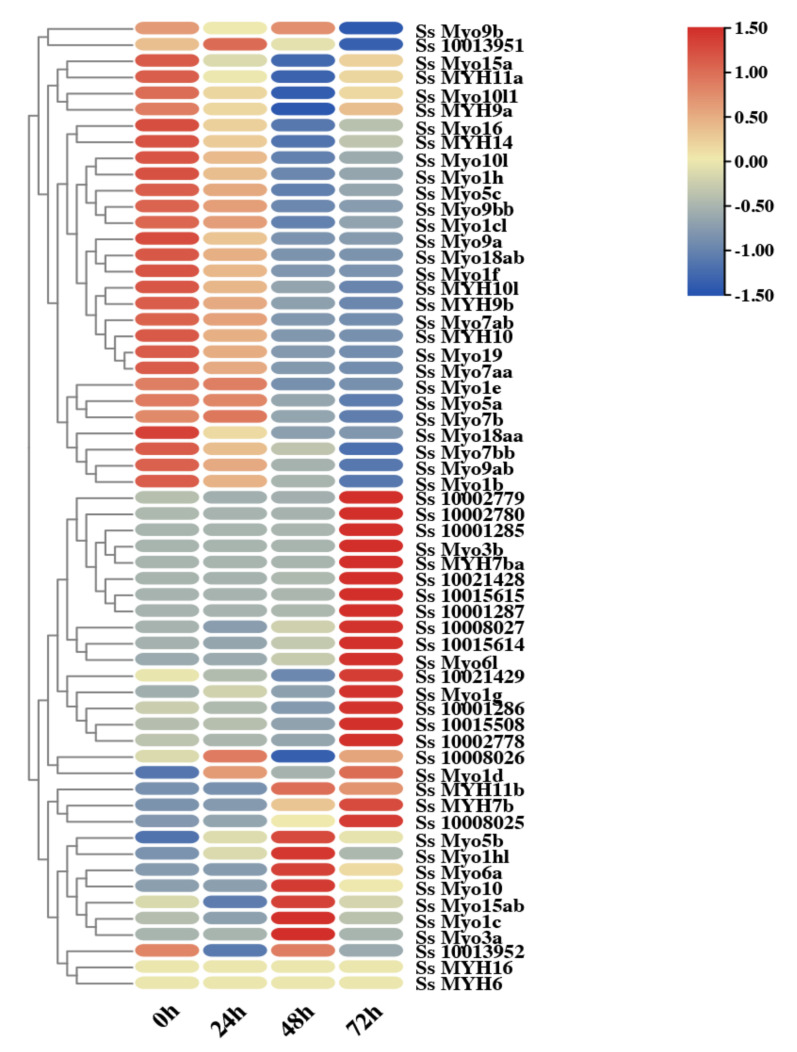
The expression patterns of *myosin* genes in muscle cell lines at different time points after differentiation induction. The color scale represented the TPM (transcripts-per-million) value. The red and blue color represented the relatively higher and lower TPM (transcripts-per-million) value, respectively.

To further investigate the roles of *myosin* genes in the muscle development, we concluded the expression patterns of *myosin* genes during the process of myoblasts differentiation. As shown in Figure 7, *myosin* genes were differently regulated during this process. *Myo1e*, *Myo3a*, *Myo5b*, and *Ss_10013951* exhibited the earlies response to differentiation treatment, showing the highest expression level at 24 h. Later on, *Myo15ab*, *Myo1f*, *Myo1c*, and *Myo6a* were up-regulated at 48 h, and then decreased. Most *Myo2* genes showed the highest expression level at 72 h after differentiation induction. However, a large number of *myosin* genes were significantly down-regulated during differentiation process. In addition, the expression levels of *Myo7aa* and *Myo1hl* did not change, suggesting that they are not involved in myoblasts differentiation.

## 4. Discussion

Skeletal muscle development and growth contribute to the size and weight of cultured animals. With the development of aquaculture industry, many strategies have been applied to promoting the muscle growth, and the genetic studies on muscle growth got more and more attention [35,36,37]. Myosin are a large and conserved family of cytoskeletal motor proteins that bind actin and participate in a broad range of biological processes [1,38]. However, little is known about *myosin* genes in teleost. In this study, we isolated and characterized 60 *myosin* family genes in *S. schlegelii* based on genome and transcriptome datasets, and initially elucidated the involvement of *myosin* genes in myoblasts differentiation and muscle growth of *S. schlegelii*.

### 4.1. Diverse Functions of Myosin Genes in S. schlegelii 

The gene expression patterns provide insight to the function and biological activity of target genes. Our results showed *myosin* genes were ubiquitously expressed in different tissues with diverse expression levels but with subfamily-specific pattern. Previous studies concluded that muscle function was determined by its structure and fiber type composition [39], so we speculated the divergent expression patterns of *S. schlegelii myosin* genes might be related to different types and compositions of muscle fibers in different tissues. Interestingly, we found the tandem duplicated *MYH2* genes of *S. schlegelii* were extremely highly expressed in skeletal muscle, suggesting their potential roles in skeletal muscle development and growth which directly determine the size of fish body. In addition, other representative *MYHs* showed conserved expression patterns between fish and mammals. For example, *MYH6* tend to be expressed in atrial muscle and *MYH7* is abundant in ventricular muscle in mammals, the mutation of *MYH6* or *MYH7* will lead to the lesions of heart [40,41,42,43]. In *S. schlegelii*, we also identified the highest expression level of *MYH7b* and *MYH6* in the heart, suggesting their potential conserved function between teleost and mammals. In addition, *MYH11b* was detected to be abundant in *S. schlegelii* intestines, which contain a lot of smooth muscle. This is consistent with the previous report that *MYH11* functions in smooth muscle in human [44]. For unconventional *myosin*, quite a few number of genes were detected the highest levels in brain and gonad of *S. schlegelii*, suggesting their potential roles in neuron and reproductive systems, as described in previous studies [45]. Taken together, the *myosin* subfamily genes may conduct conserved functions between human and teleost. Most *myosin* genes started to be highly expressed at pre-hatching stage, suggesting the muscle fibers synthesis was significantly active during this period. 

### 4.2. Expansion of Myo2 Subfamily and Their Involvement in Skeletal Muscle Growth in S. schlegelii

Teleost had undergone the TGD during the evolution, resulted in two or more copies of genes existing in teleost genome [46]. Generally, gene expansion results in strengthened phenotype and drives the evolutionary process of adaption [47]. In *S. schlegelii*, we identified and characterized 60 *myosin* genes, containing 27 conventional *myosin* genes, which belong to *Myo2* subfamily. Compared to other teleost species, *S. schlegelii Myo2* subfamily showed significant expansion (Figure 1), which was driven by the tandem duplication events (Figure 4). Previous studies have shown massive expansion of gene family in teleost might be related to the vital roles of family members in certain biological processes. For example, the expansion of NLR family suggest that NLRs have a more substantial role in the innate immunity in haddock [48]. The expansion of *tdrd* family may be related to their important function in germline in Japanese flounder [49]. Members of *Myo2* subfamily have been identified to play an important role in muscle development in teleost but in different regulatory ways. For example, in torafugu, *MYH_M86-1_* and *MYH_86-2_* were reported to have markedly different expression patterns in muscle [20]. Three embryonic *MYHs* were located in the same or different cranial muscles of common carp larvae [50]. In *S. schlegelii*, some of *MYH2* genes generated by tandem duplication showed distinct expression patterns. As the marker of mature skeletal muscle differentiation, most *MYH2* genes expressed highly in skeletal muscle whereas several *MYH2* genes such as *Ss_10002778* and *Ss_10002779* expressed highly in testis, *Ss_10021428* highly in gill, *Ss_10013951* and *Ss_10013952* highly in brain, suggesting the expanded *MYH2* genes might gain the neo-function in smooth muscle and gland cells instead of the traditional role in skeletal muscle. This result provides the evidence that gene duplication is the important source for the emergence of evolutionary novelties and neofunctionalization of gene families. Taken together, we speculate that the expansion of *Myo2* subfamily genes contribute to development and growth of both skeletal muscle and some other important organs in *S. schlegelii*. The specific roles for each member need further observation. 

We selected five skeletal muscle-highly-expressed *MYH* genes and analyzed their expression levels in the fast-growth stages of juvenile and adult *S. schlegelii*. Our current results showed the expression levels of *MYHs* were significantly up-regulated from 50 dpp or 75 dpp, corresponding to the timepoints of significant increase of number and size of muscle fibers. In juvenile torafugu, *MYHs* was confirmed to be involved in mosaic hyperplasia and stratified hyperplasia, which contributed to the juvenile skeletal muscle growth [20], the similar conclusion was also obtained in some other species, such as shrimp, carp, and Atlantic cod [15,51,52]. In adult *S. schlegelii*, the selected genes showed significantly higher expression in 2.5-year-old than in 1.5-year-old fish, suggesting more active synthesis of muscle fibers in 2.5-year-old. This is in accordance with our recent finding that 2.5-year-old fish grows faster than 1.5-year-old fish (the details of growth pattern will be reported separately). To sum up, *Myo2* genes are involved in skeletal muscle fibers development and contribute to the skeletal muscle growth of juvenile and adult *S. schlegelii*.

### 4.3. Myosin Genes Participate in Myoblast Differentiation of S. schlegelii

*Myosin* genes of *S. schlegelii* were identified to be involved in muscle fiber growth in our studies; however, the mechanisms by which step *myosin* is involved in muscle development is unclear. As described in previous studies, myoblast proliferation and differentiation promotes the formation of muscle fibers, and then contributes to the growth of skeletal muscles in cultured animals [53]. The culture and differentiation induction of *S. schlegelii* myoblast in vitro, and the availability of transcriptome data at different timepoints during myoblast differentiation give us a chance to analyze the involvement of *myosin* genes during myoblast differentiation. As our results showed, *myosin* genes displayed quite different response in the process of myoblast differentiation. Most *Myo2* subfamily genes were upregulated in this process, especially the *MYH2* genes cluster, which was identified significantly expanded and highly expressed in the skeletal muscle in *S. schlegelii*. As the late differentiation marker [54], it is easy to understand the up-regulation of *MYH2* with the differentiation process of myoblast cells in *S. schlegelii*, which has also been widely demonstrated in other species [55,56,57].

However, quite a few of *myosin* genes, containing both conventional and unconventional *myosin*, were down-regulated during cell differentiation. For muscle fiber development and growth, except for the contribution of increased protein synthesis and increased cell size due to cell differentiation, the formation of new myoblast by cell proliferation also plays a vital role [58]. It has been widely identified that *myosin* could be involved in cell proliferation. For example, knockdown of *myosin 6* inhibits proliferation of hepatocellular carcinoma cells and oral squamous cell carcinoma cells [59,60]. *myosin 16*, an unconventional member, is proved to have a role in regulation of cell cycle and cell proliferation [61]. In *Drosophila*, on-muscle *myosin 2* is required for cell proliferation during wing morphogenesis [62]. In our study, the down-regulation of *myosin* during cell differentiation suggest their potential roles in cell proliferation in *S. schlegelii*. Taken together, *S. schlegelii myosin* family genes participate in skeletal muscle growth by involvement both in myoblast proliferation and differentiation.

## 5. Conclusions

We identified and characterized 60 *myosin* family genes in *S. schlegelii* and demonstrated the expansion of *myo2* subfamily in this species. The expression profiling of *myosin* provides the evidence that they are involved in different types and compositions of muscle fibers in different tissues. Moreover, the expanded *myo2* genes actively participate in the skeletal muscle growth both in juvenile and adult *S. schlegelii*. During myoblast differentiation, *myosin* responded differently, revealing their divergent functions in myoblast proliferation and differentiation. Taken together, our work characterized *myosin* genes systemically and demonstrated their diverse functions in a marine teleost species. This lays foundation for the further studies of muscle growth regulation and molecular mechanisms of indeterminate skeletal muscle growth of large teleost fishes. 

## Figures and Tables

**Table 1:** The detailed information of *myosin* genes in *S. schlegelii*. Abbreviations for protein locations are: N, nucleus; M, mitochondria; C, cytoplasm; Es, extracellular space; Cs, cytoskeleton.

Gene	Chromosome	CDS (bp)	Amino Acid (aa)	Mw (Kda)	PI	Location	Number of Transcript
*Myo1b*	Chr 20	3513	1171	135.52	9.43	C, N, M, Cs	4
*Myo1c*	Chr 4	2928	976	112.43	9.23	C, N, M, Cs, Es	4
*Myo1cl*	Chr 12	3204	1068	122.32	8.98	C, N, Cs, Es	1
*Myo1d*	Chr 18	3528	1176	134.19	9.3	C, N, M	3
*Myo1e*	Chr 2	3297	1099	124.93	9.21	Es, M	1
*Myo1f*	Chr 6	3219	1073	122.63	9.23	C, N, M, Cs	3
*Myo1g*	Chr 11	3033	1011	115.55	7.96	C, N, M, Cs	1
*Myo1h*	Chr 8	3090	1030	118.74	8.6	C, N, M	3
*Myo1hl*	Chr 17	3102	1034	119.84	9.13	C, N, M	1
*Myo3a*	Chr 21	5112	1704	194.21	6.52	C, N, M	2
*Myo3b*	Chr 20	4017	1339	152.55	8.45	C, N, M, Cs	2
*Myo5a*	Chr 2	5577	1859	214.27	8.84	C, N, M	3
*Myo5b*	Chr 6	4662	1554	176.03	9.12	C, N, M	1
*Myo5c*	Chr 9	10059	3353	388.34	8.43	C, N, M	1
*Myo6*	Chr 14	2862	954	109.7	8.59	C, N, M, Cs	1
*Myo6l*	Chr 22	3273	1091	125.21	8.47	C, N, M	2
*Myo7aa*	Chr 11	6330	2110	243.81	8.92	C, N	3
*Myo7ab*	Chr 4	5940	1980	228.49	8.88	C, N, M	1
*Myo7b*	Chr 6	6696	2232	256.07	8.88	C, N	7
*Myo7bb*	Chr 7	6252	2084	237.81	8.16	C, N	2
*Myo9aa*	Chr 2	7689	2563	291.95	8.45	C, N	4
*Myo9ab*	Chr 9	7224	2408	274.34	8.58	C, N	7
*Myo9ba*	Chr 6	7464	2488	282.91	7.63	C, N	3
*Myo9bb*	Chr 7	5862	1954	223.1	8.6	C, N, Cs	5
*Myo10*	Chr 20	5763	1921	220.93	5.57	C, N	1
*Myo10l*	Chr 14	6030	2010	227.9	6.8	C, N	2
*Myo10l1*	Chr 7	5745	1915	218.78	6.13	C, N	2
*Myo15a*	Chr 5	9417	3139	352.11	9.02	C, N, M	1
*Myo15ab*	Chr 13	10,929	3643	406.73	8.35	C, N	1
*Myo16*	Chr 20	5730	1910	210.75	8.41	C, N	2
*Myo18aa*	Chr 4	8160	2720	304.89	6.78	C, N	6
*Myo18ab*	Chr 12	6903	2301	259.71	6.82	C, N	5
*Myo19*	Chr 4	2913	971	110.4	8.27	C, N, M, Es	1
*Ss MYH6*	Chr 22	5802	1934	222.63	5.62	C, N, Cs, Es	1
*Ss MYH7ba*	Chr 10	5712	1904	219.7	6.19	C, N, M, Cs	1
*MYH7bb*	Chr 1	5850	1950	224.95	5.95	C, N, Cs	1
*Ss MYH9a*	Chr 5	5904	1968	228.58	5.26	C, N, M, Cs	1
*Ss MYH9b*	Chr 13	5928	1976	229.45	5.5	C, N, M, Cs	1
*Ss MYH10*	Chr 5	5985	1995	231.74	5.42	C, N, M, Cs	2
*Ss MYH10l*	Chr 13	5793	1931	223.77	5.46	C, N, M, Cs	2
*Ss MYH11a*	Chr 5	5916	1972	227.02	5.43	C, N, M, Cs	2
*Ss MYH11b*	Chr 13	5850	1950	224.29	5.48	C, N, M, Cs	1
*Ss MYH14*	Chr 19	5763	1921	220.93	5.57	C, N, M	4
*Ss MYH16*	Chr 13	5760	1920	220.35	5.86	C, N, M, Cs	1
*Ss 10008025*	Chr 7	11,313	3771	435.08	5.92	C, N, Cs, Es	1
*Ss 10008026*	Chr 7	5877	1959	225.07	5.78	C, N, Cs, Es	1
*Ss 10008027*	Chr 7	5823	1941	223.39	5.8	C, N, Cs, Es	1
*Ss 10001285*	Chr 9	4020	1340	154.38	5.57	C, N, M, Cs	1
*Ss 10001286*	Chr 9	5817	1939	221.86	5.8	C, N, Cs, Es	1
*Ss 10001287*	Chr 9	5664	1888	216.24	5.49	C, N, Cs, Es	2
*Ss 10002778*	Chr 1	5751	1917	219.67	5.58	C, N, M, Cs	1
*Ss 10002779*	Chr 1	5937	1979	227.43	5.86	C, N, M, Cs	4
*Ss 10002780*	Chr 1	5811	1937	222.69	5.75	C, N, M, Cs	1
*Ss 10013951*	Chr 13	5787	1929	222.94	5.78	C, N, M, Cs	1
*Ss 10013952*	Chr 13	5736	1912	220.14	5.66	C, N, M, Cs	1
*Ss 10015508*	Chr 9	5571	1857	212.53	5.66	C, N, M, Cs	5
*Ss10015614*	Chr 9	5886	1962	224.78	5.58	C, N, M, Cs	1
*Ss 10015615*	Chr 9	5829	1943	222.54	5.5	C, N, M, Cs	1
*Ss 10021428*	Chr 13	5778	1926	220.78	5.76	C, N, M, Cs	3
*Ss 10021429*	Chr 13	5841	1947	223.01	5.67	C, N, M, Cs	1

**Table 2:** The tandem duplication events of *myosin* genes identified in *S. schlegelii*.

Cluster Number	Gene ID	Chromosome	Start Site	End Site
1	Ss_10021428	13	9553704	9568469
	Ss_10021429	13	9589690	9601364
2	Ss_10015614	9	11373314	11383630
	Ss_10015615	9	11388379	11398341
3	Ss_10013951	13	17872608	17905057
	Ss_10013952	13	17911150	17926242
4	Ss_10008025	7	29692553	29729078
	Ss_10008026	7	29738637	29750296
	Ss_10008027	7	29758471	29783643
5	Ss_10002778	1	34544361	34556333
	Ss_10002779	1	34564613	34580184
	Ss_10002780	1	34589861	34601724
6	Ss_10001285	9	11434203	11445087
	Ss_10001286	9	11448018	11458669
	Ss_10001287	9	11466155	11477014

## Data Availability

The datasets of whole genome sequencing of *S. schlegelii* and RNA-seq of tissues and developmental stages were available in CNSA (CNGB Nucleotide sequence archive) with the accession ID CNP0000222. The RNA-seq dataset of *S. schlegelii* myoblasts differentiation was submitted to NCBI SRA (BioProject ID: PRJNA661185).

## Supplementary Materials

The following are available online at https://www.mdpi.com/article/10.3390/genes12060808/s1, Figure S1. Synteny analysis of myosins and adjacent genes among spotted gar (Lepisosteus oculatus), medaka (oryzias latipes), threespine stickleback (Gasterosteus aculeatus), human (Homo sapiens) and mouse (Mus musculus). Orthologs of each gene were shown in the same color. Gene orientation is indicated by the direction of the arrows. Table S1: The TPM values of Sebastes Schlegelii myosins in different tissues. Table S2: The TPM values of Sebastes Schlegelii myosins during different developmental stages. Table S3: The TPM values of Sebastes Schlegelii myosins during myoblast differentiation in vitro.
