# Supplementary figures and images for: Genome-Wide Identification, Characterization and Expression Profiling of myosin Family Genes in Sebastes schlegelii
Source: Genes (Basel). 2021 May 25;12(6):808. doi: 10.3390/genes12060808 (PMC8228858; doi:10.3390/genes12060808)

1

*Myo1c*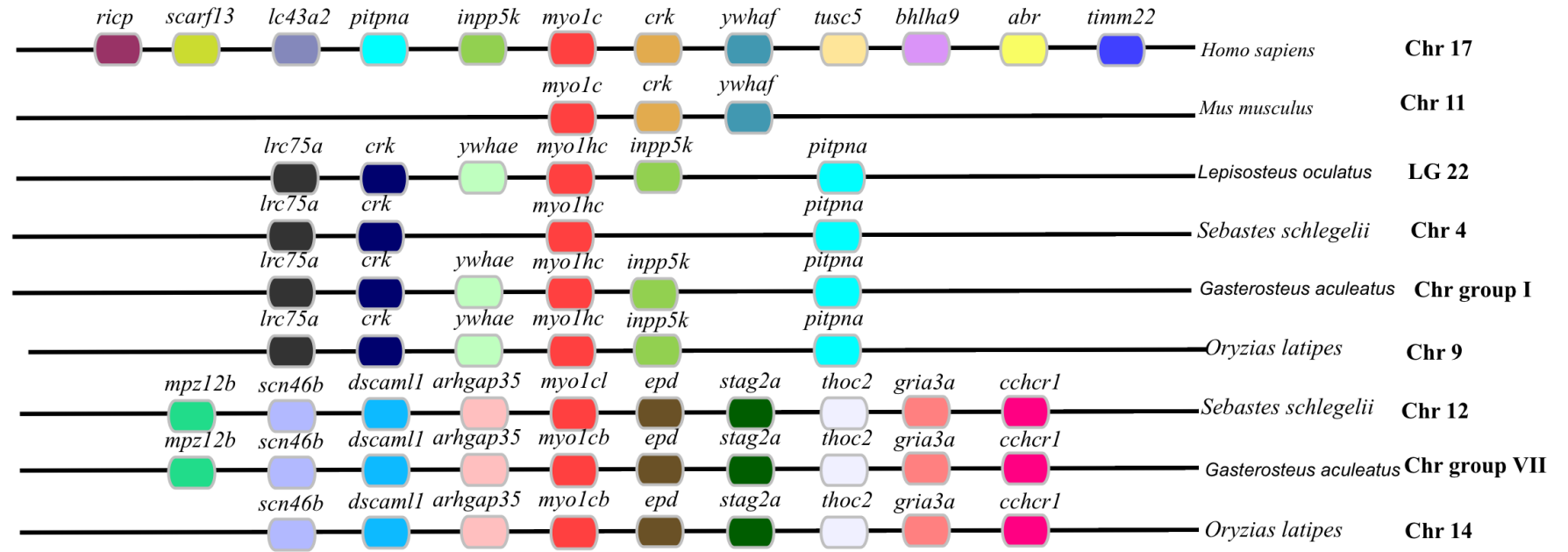

2

*Myo1h*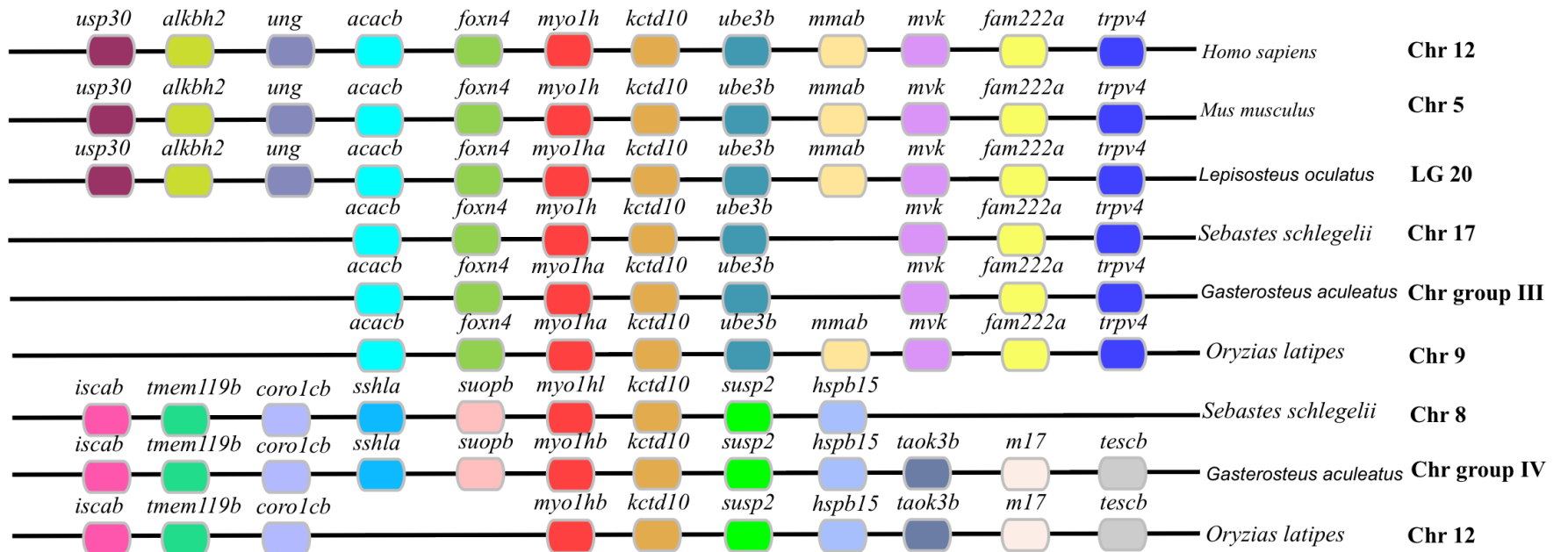

Myo6

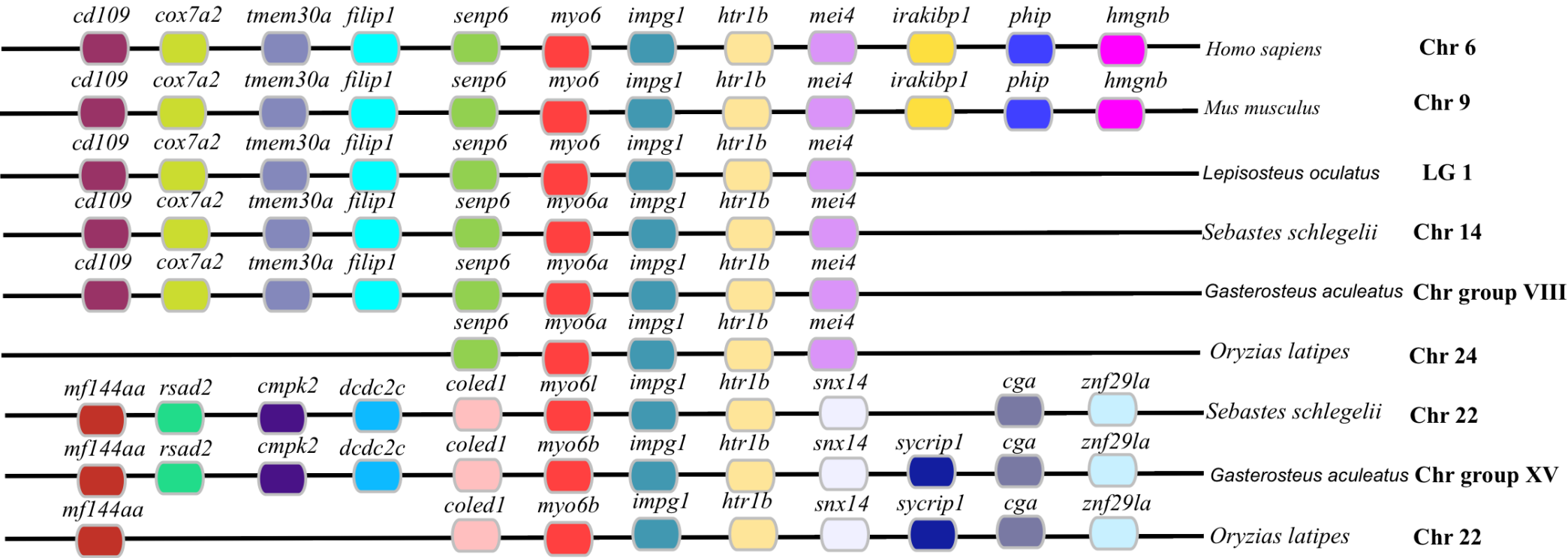

4

**Myo 7a**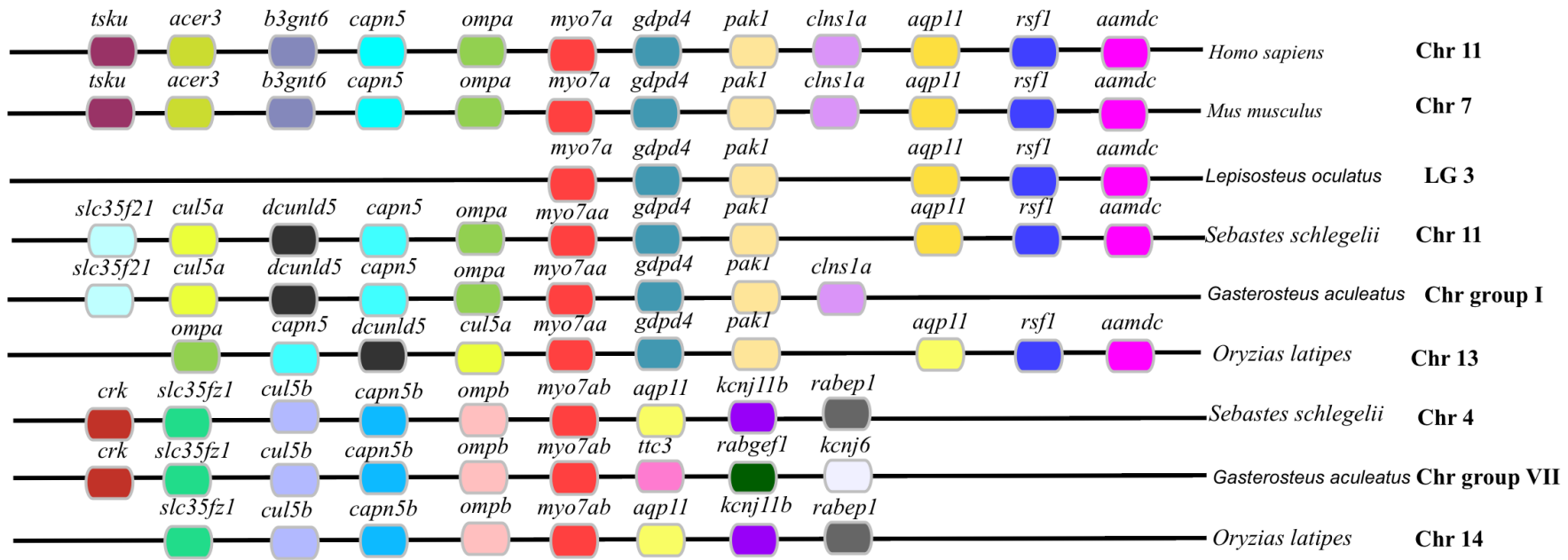

5

**Myo 7b**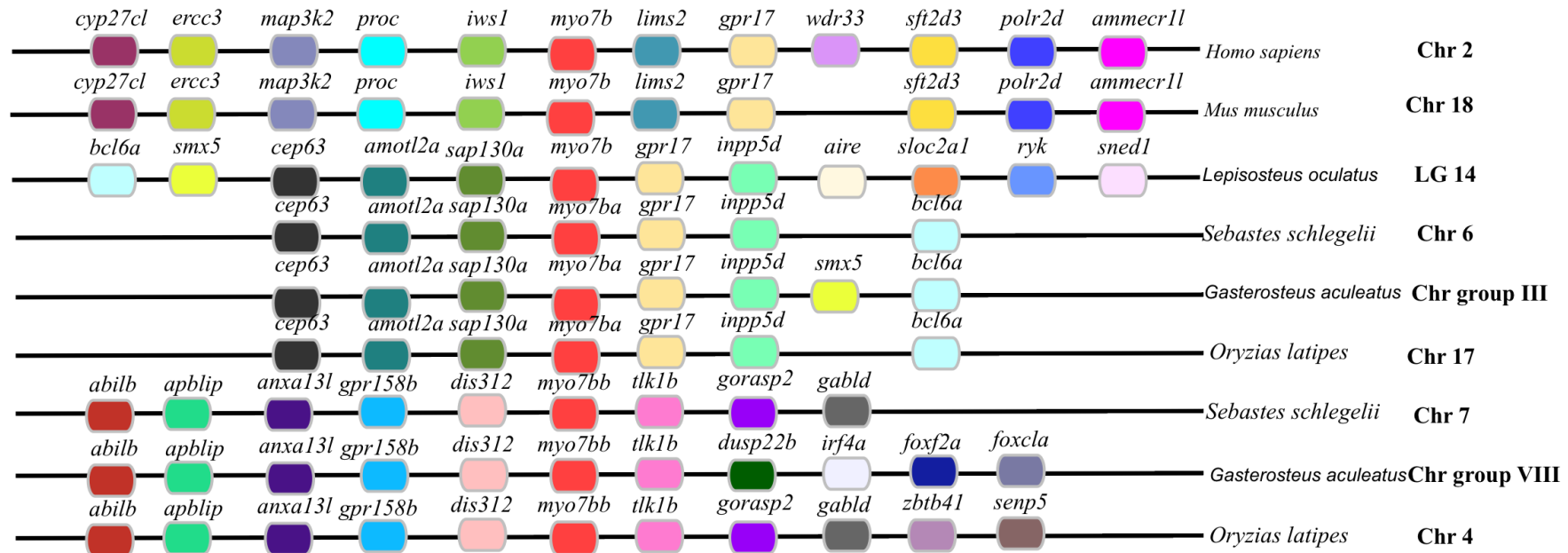

6

*Myo9a*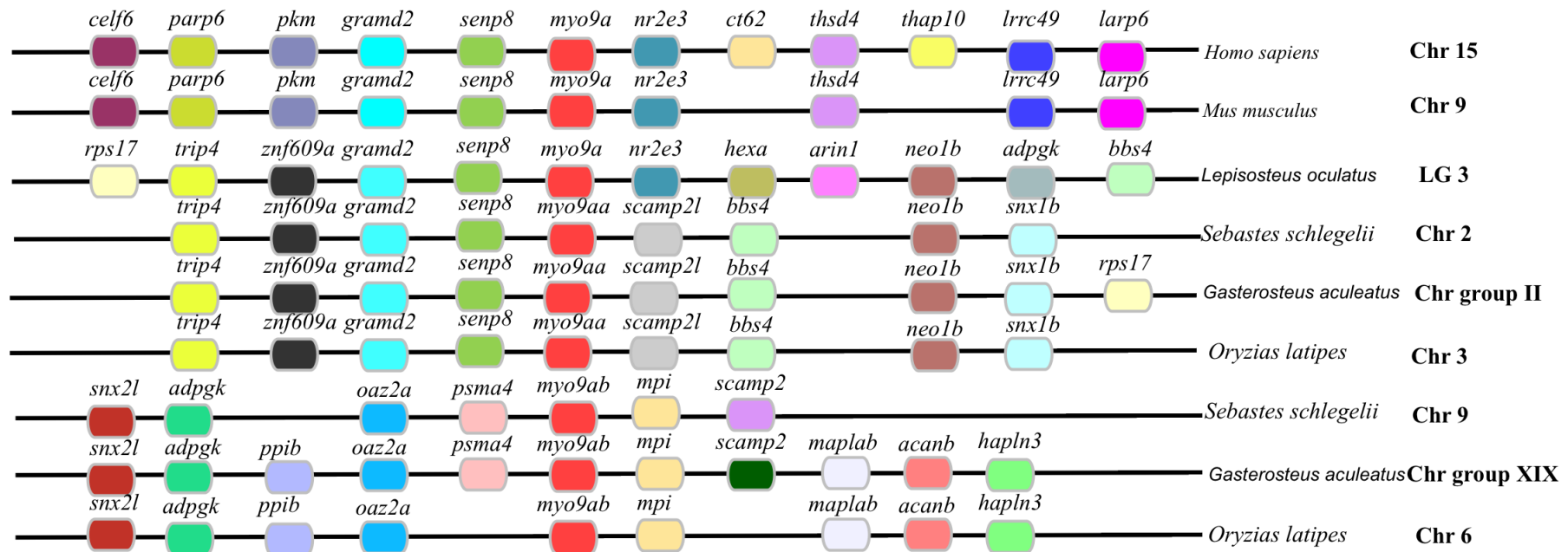

7

*Myo9b*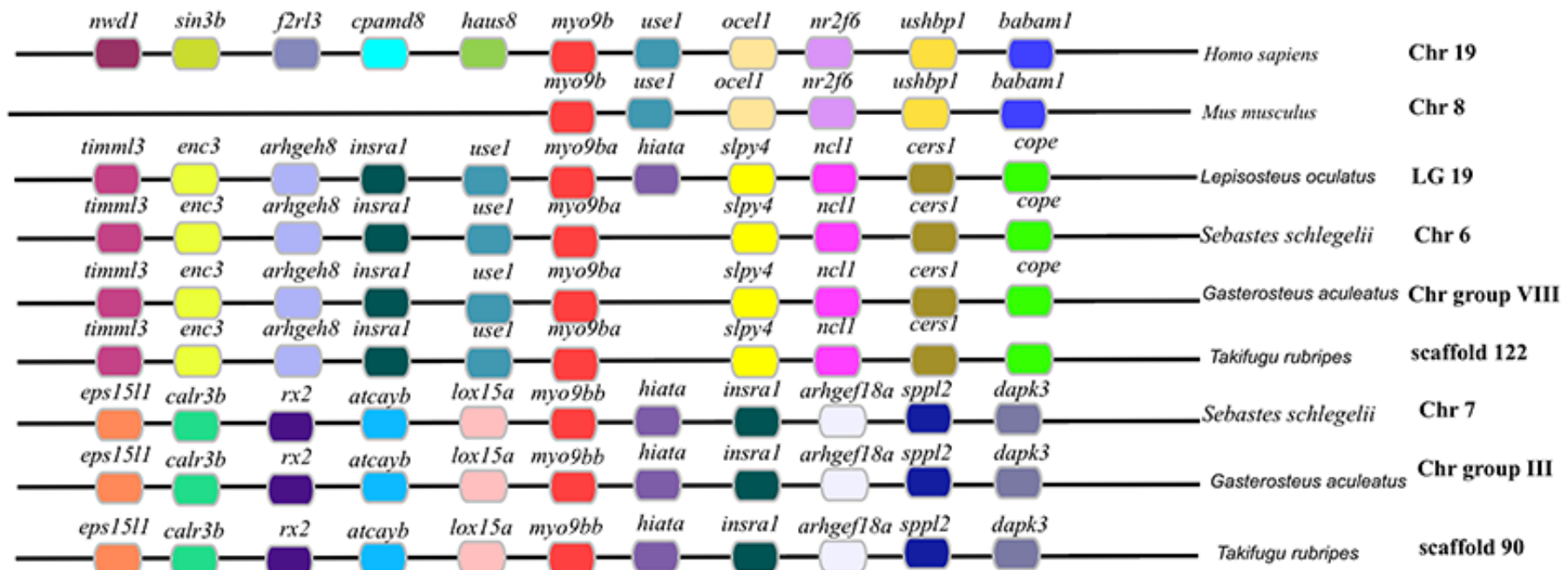

Myo10

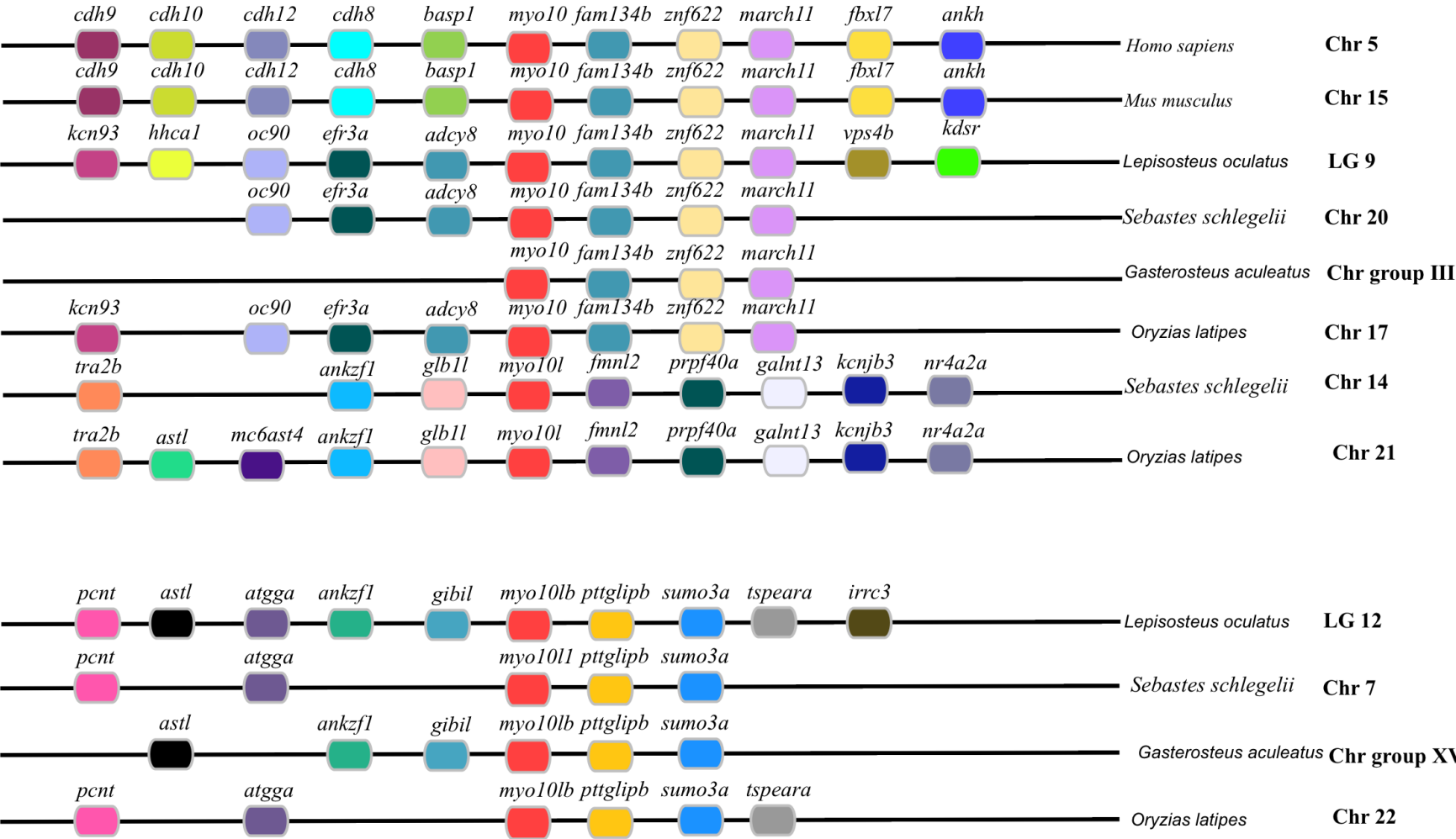

9

**Myo15a**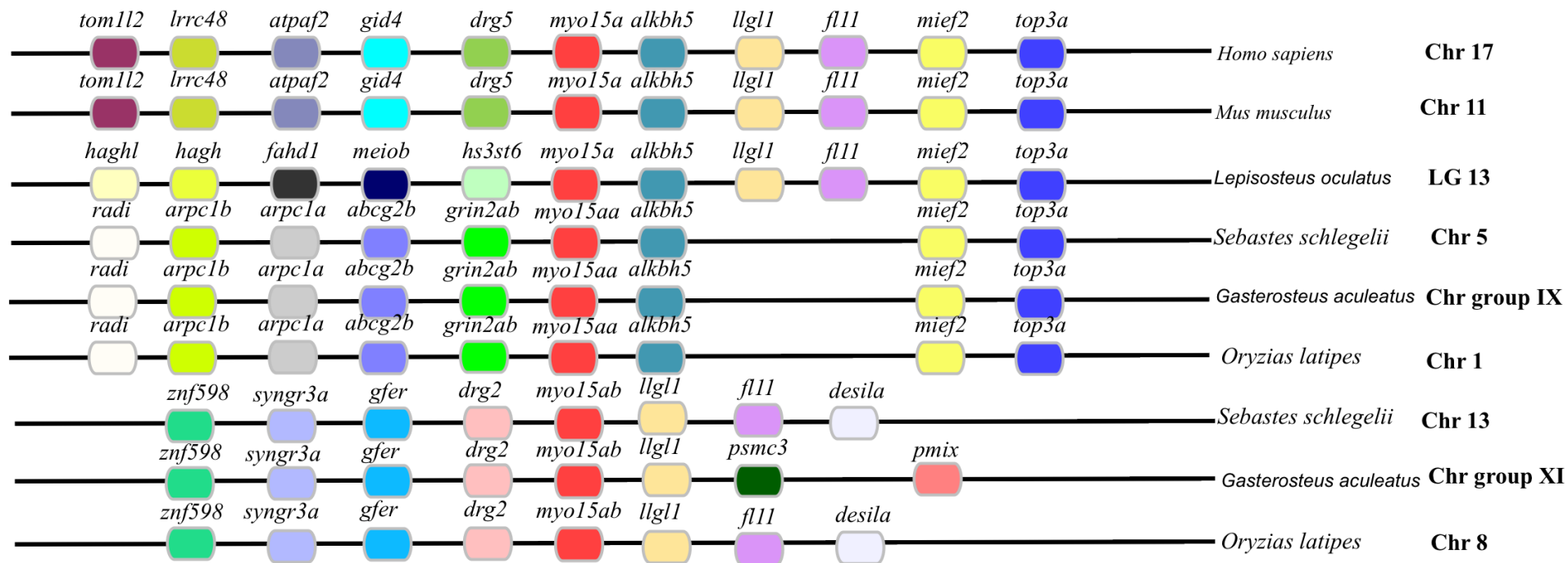

10

**Myo18a**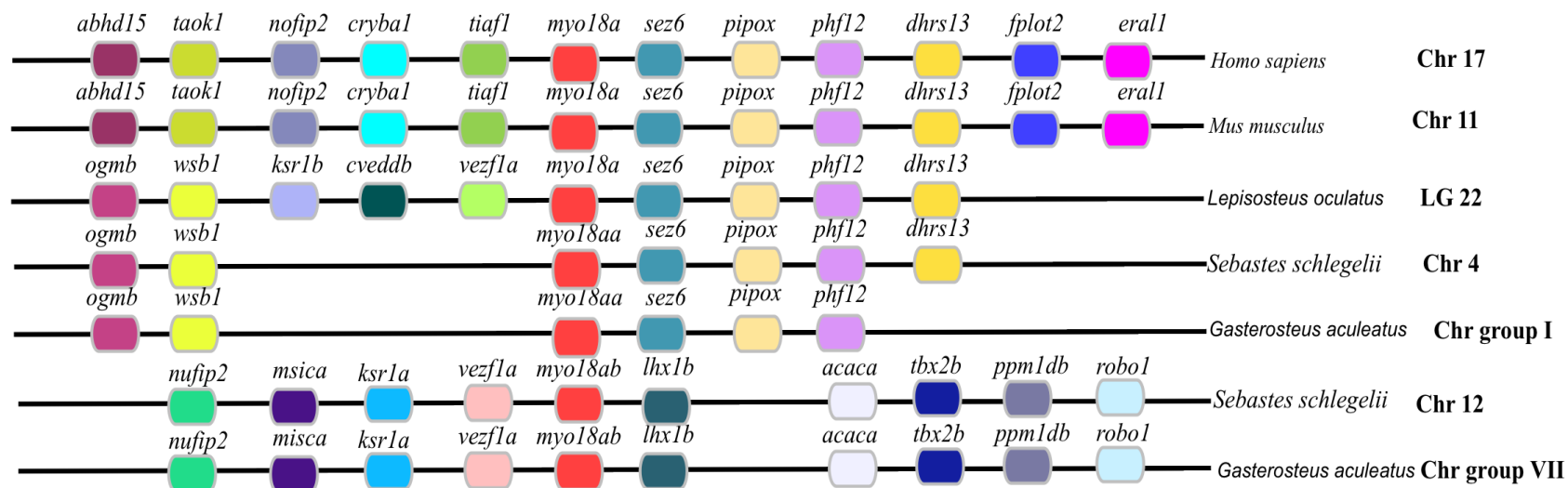

11

Myh9

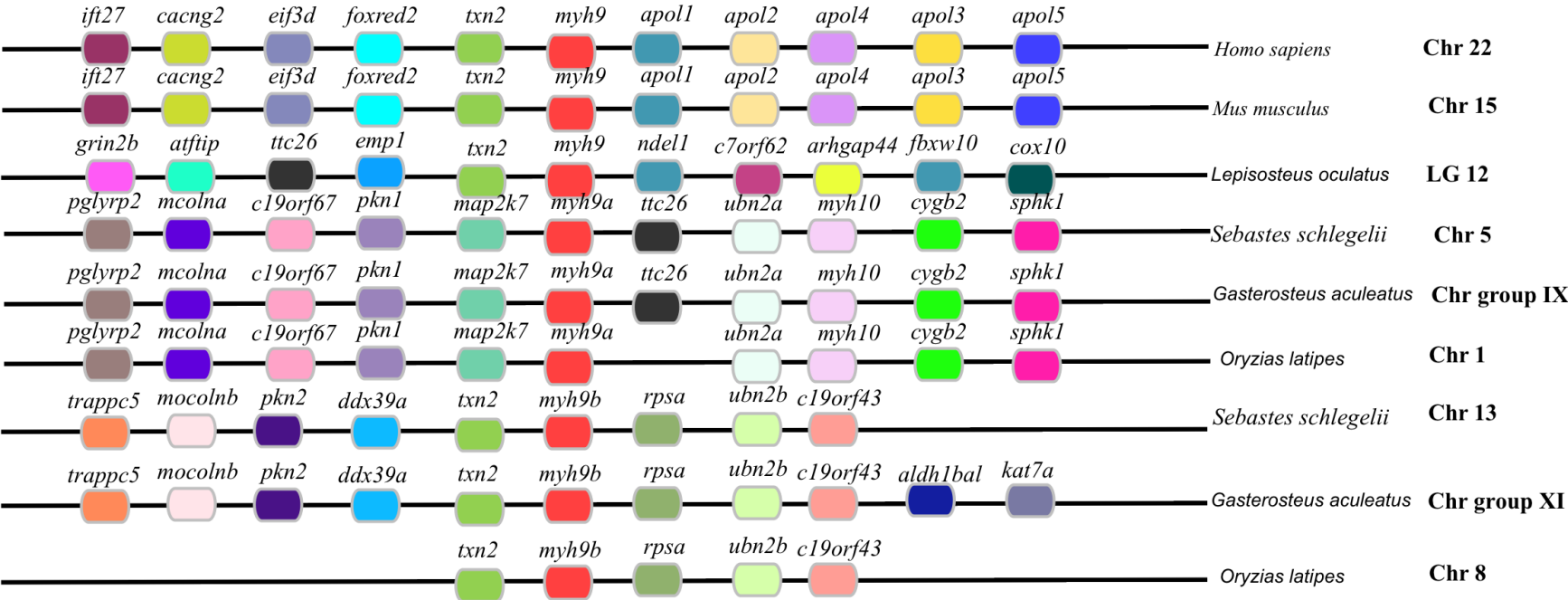

12

Myh10

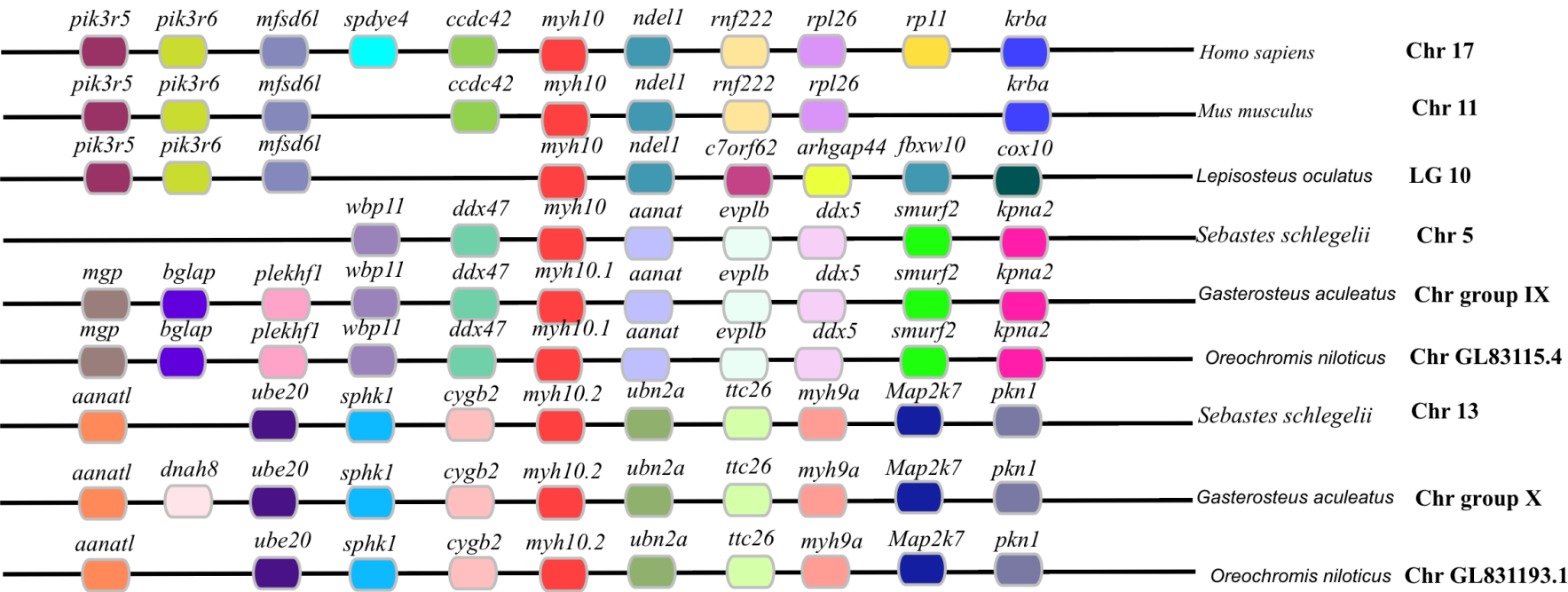

Myh11

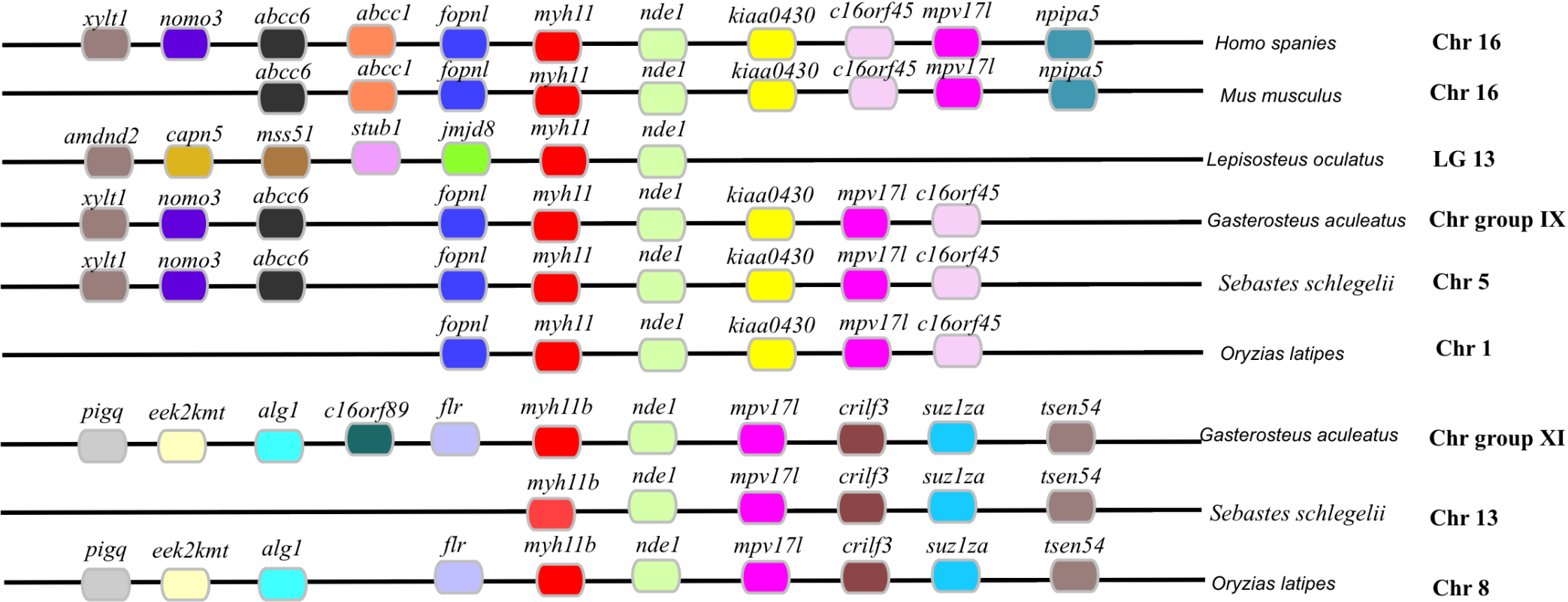

Myh 7b

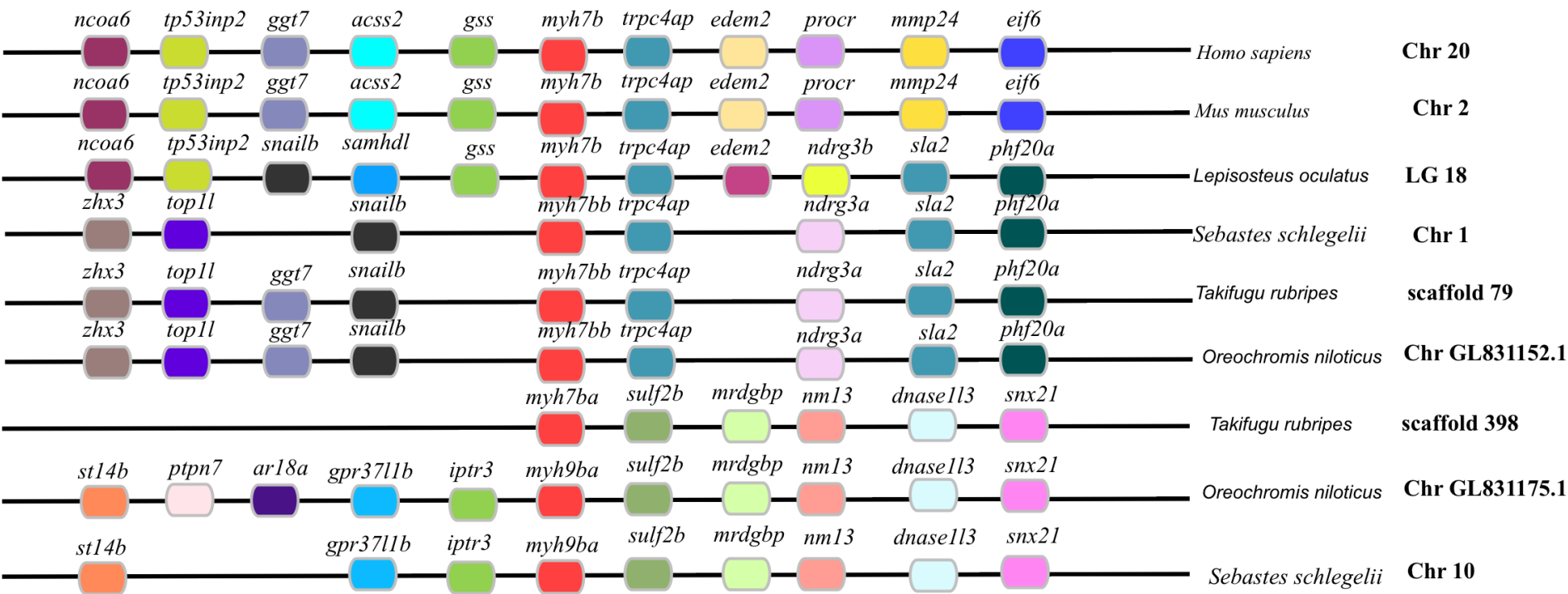

Myh 7

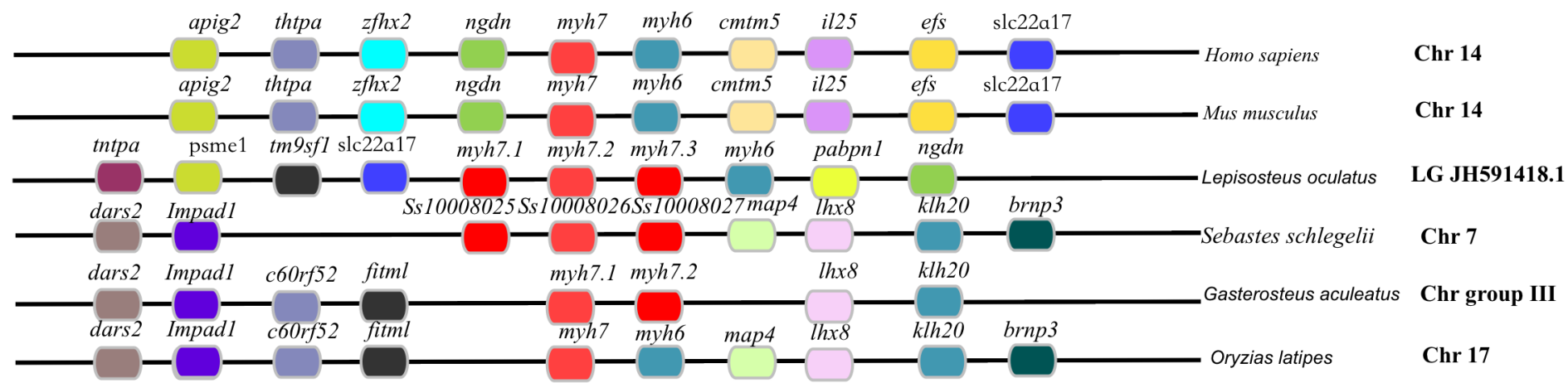

*Myh2*

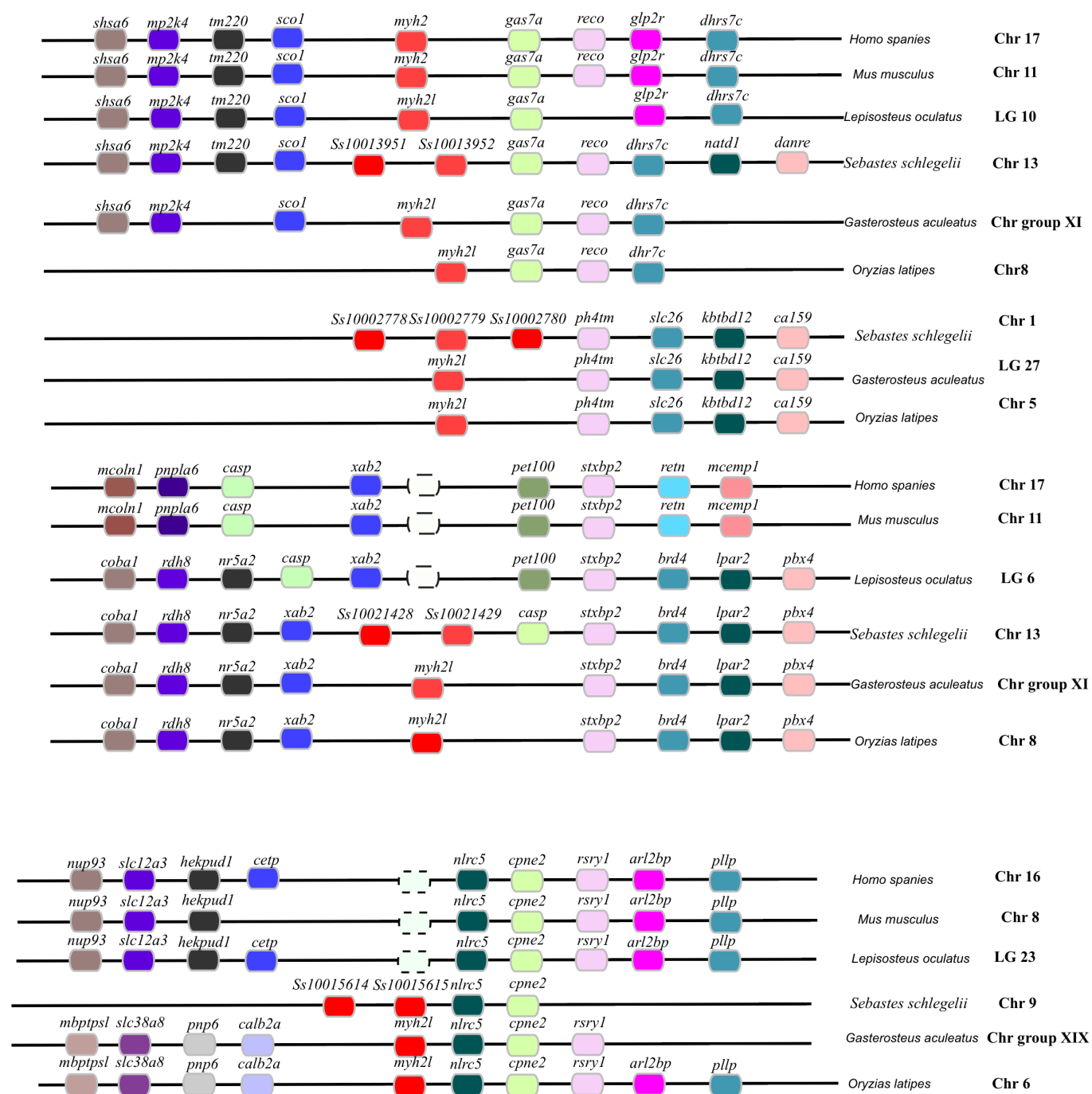

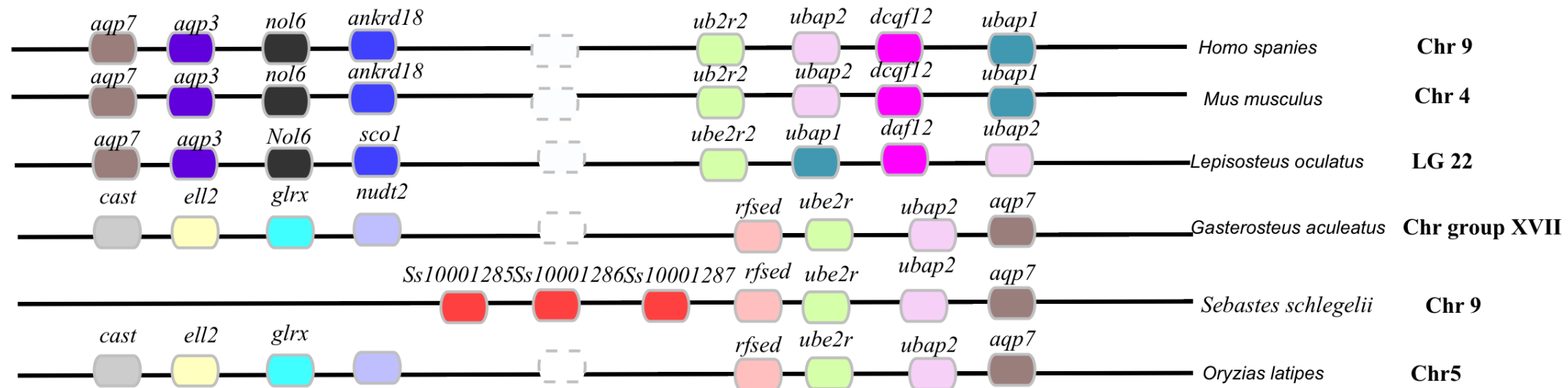

Supplement: Supplementary file 1 [file genes-12-00808-s001.zip › genes-1202531 supplementary/genes-1202531 supplementary.pdf]
